# LncRNA FAM83A-AS1 promotes ESCC progression by regulating miR-214/CDC25B axis

**DOI:** 10.7150/jca.54007

**Published:** 2021-01-01

**Authors:** Jinlin Jia, Hongle Li, Jie Chu, Jinxiu Sheng, Chang Wang, Zimo Jia, Weiwei Meng, Huiqing Yin, Junhu Wan, Fucheng He

**Affiliations:** 1 Department of Medical Laboratory, The First Affiliated Hospital of Zhengzhou University, Zhengzhou, Henan Province, China.; 2 Department of Molecular Pathology, The Affiliated Cancer Hospital of Zhengzhou University, Zhengzhou, Henan Province, China.; 3 Department of Clinical Medicine, Hebei Medical University, Shijiazhuang, Hebei Province, China.; 4 Department of Blood Transfusion, The Second Affiliated Hospital of Zhengzhou University, Zhengzhou, Henan Province, China.

**Keywords:** FAM83A-AS1, lncRNA, ESCC, miR-214, CDC25B

## Abstract

**Background:** Recent researches have pinpointed that long non-coding RNA (lncRNA) was tightly related to the carcinogenesis. However, the function of lncRNA in esophageal cell squamous carcinoma (ESCC) remains to be explored. In the current study, we assessed the expression pattern and the biological function of FAM83A-AS1 in ESCC.

**Methods:** qRT-PCR was used to detect the expression of FAM83A-AS1, miR-214, and CDC25B expression in ESCC tissues and cell lines. CCK-8, transwell, apoptosis and cell cycle assays were performed to define the function of FAM83A-AS1 in ESCC cells. Furthermore, the regulation of miR-214 by FAM83A-AS1 was defined by qRT- PCR and rescue assays. In addition, the association between CDC25B, miR-214, CDC25B was confirmed by qRT-PCR.

**Results:** Here, we discovered that FAM83A-AS1 was strongly expressed in ESCC tissues. FAM83A-AS1 abundance was associated with TNM stages and the differentiation grade of ESCC patients. The receiver operating characteristic curve (ROC) analysis indicated the high accuracy of FAM83A-AS1 in ESCC diagnosis. Functionally, inhibiting FAM83A-AS1 repressed cell proliferation, migration, and invasion in ESCC. In addition, we found that FAM83A-AS1 accelerated the cell cycle while inhibited cell apoptosis. Mechanistically, we found that FAM83A-AS1 regulated miR-214 expression, and there was a negative correlation between miR-214 and FAM83A-AS1 in ESCC. Rescue assay indicated that miR-214 could impair the suppressing effect of cell migration induced by FAM83A-AS1 depletion. Furthermore, CDC25B was a direct target of miR-214, and FAM83A-AS1 enhanced CDC25B expression while miR-214 positively CDC25B expression in ESCC.

**Conclusions:** Collectively, we concluded that FAM83A-AS1 facilitated ESCC progression by regulating the miR-214/CDC25B axis. Our study showed FAM83A-AS1 may act as a promising target for ESCC diagnosis and therapy.

## Introduction

Esophageal cancer (EC) is one of the most common malignancies worldwide, which is always accompanied by high morbidity and mortality 1. Approximately 80% of EC is clarified as esophageal cell squamous carcinoma (ESCC) in China 2. Despite greatly advanced progress that has been achieved in the diagnosis and treatment of ESCC over the past decades, the high occurrence and poor survival of ESCC remains a public health concern 3,4, and the molecular mechanism of ESCC development remains to be largely elucidated. Therefore, it is urgent to find out the precise molecular mechanism underlying ESCC pathogenesis. Detailed investigations can provide novel sights into the effective therapeutic strategy for ESCC.

Recently, increasing attention has been paid to long non-coding RNA (lncRNA) because of their diverse effect in the hallmarks of cancer 5. LncRNA is a kind of non-coding transcript, whose length is longer than 200 nucleotides (nt), and lncRNA can participate in numerous biological processes that involved in the tumorigenesis and metastasis procedure of ESCC 6. It is established that lncRNA functions through epigenetic, transcriptional and post-transcriptional mechanisms 7,8. Emerging studies have highlighted that lncRNA could serve as a miRNA sponge to regulate downstream gene expression and activity 9,10. The competing endogenous RNA (ceRNA) mechanism has been widely recognized as an important and common working model of lncRNA. For instance, MNX1-AS1 promoted cell growth and metastasis procedure by binding to miR-34a in ESCC 11. LEF1-AS1 served as the sponge of miR-489-3p and increased the HIGD1A expression level, ultimately accelerating the malignancy phenotype of glioma 12. Though many lncRNAs have been defined as crucial modulators in cancer, the association between lncRNA and ESCC pathogenesis remains largely unclear.

FAM83A-AS1 was reported to exert oncogenic function in lung cancer 13. FAM83A-AS1 was significantly increased in lung cancer tissues and FAM83A-AS1 enhanced cell proliferation and migration potential by targeting the miR-150-3p/MMP14 axis 14. Besides, FAM83A-AS1 facilitated tumorigenesis through interacting with NOP58 to increase the FAM83A stability in hepatocellular carcinoma 15. Herein, we found FAM83A-AS1 was dramatically overexpressed in ESCC tissues. FAM83A-AS1 expression level was associated with differentiation grade and advanced stages of ESCC patients. In addition, there was a high accuracy of FAM83A-AS1 in ESCC diagnosis. We next investigated the effects of FAM83A-AS1 in ESCC progression. FAM83A-AS1 promoted cell proliferation, migration and invasion. We also observed that FAM83A-AS1 increased the count of ESCC cells in the S phase. Notably, we identified that FAM83A-AS1 could function as the molecular sponge to regulate ESCC progression through controlling the miR-214/CDC25B axis. These findings showed that FAM83A-AS1 was a promising target of diagnosis and treatment in ESCC, which deepened our understanding of ESCC carcinogenesis.

## Materials and methods

### Tissues specimens

ESCC tissues and matched adjacent non-tumor esophageal tissues were obtained from 51 patients who were newly diagnosed and histologically confirmed ESCC by more than three pathologists at the Department of Pathology, the First Affiliated Hospital of Zhengzhou University during 2019. Fresh cancer tissues and matched adjacent non-tumor tissues were immediately collected after surgery and stored at -80 °C in RNA later until RNA derivation. Then lncRNA, miRNA, and mRNA expression levels were detected with qRT-PCR assays. Meanwhile, we collected the clinical features of these 51 participants for further analysis. All enrolled patients in this research signed written informed consent and the study project got the approval of the ethics committee of the First Affiliated Hospital of Zhengzhou University.

### Cell lines

KYSE30 and EC109 cells were obtained from the Shanghai Institute of life science cell bank center (Shanghai, China). ESCC cell lines were maintained in DMEM (Hycolne, USA) adding 10% fetal bovine serum (Hyclone, USA) along with 1% penicillin-streptomycin. Besides, cells were routinely cultured in an atmosphere of 5% CO_2_ at 37 °C and stored with cell saving (New Cell & Molecular Biotech, Suzhou, China). Cell culture dishes were acquired from Hangzhou Xinyou Biotechnology Co., Ltd.

### RNA extraction and qRT-PCR

Total RNAs isolated from ESCC tissues and cell lines were obtained with Trizol reagent (Takara, Dalian, China) according to the manufacturer's protocol. The quality of RNA was assessed by the NanoDrop 2000 spectrometer. The reverse transcription reaction was implemented by the PrimeScript™RT reagent Kit with gDNA Eraser (TakaRa, Dalian, China) to acquire the cDNAs of lncRNA and mRNA. The reverse transcription reaction of miRNA was performed with the miRNA 1st Strand cDNA Synthesis Kit (Vazyme, Nanjing, China). qRT-PCR was used to detect the relative expression of lncRNA, miRNA, and mRNA in the QuautStudio-5 system. β-Actin or U6 were defined as the endogenous controls for normalization. We adopted the 2^-△△ct^ method to analyze the differences. The primer sequence used in this research were listed: FAM83A-AS1-F: 5'-GCCCCTTCTTCTGGTTGT-3', FAM83A-AS1-R: 5'-CACCTATGCTGGGGTCAG-3'; miR-214-F: 5'-ATATCCGACAGCAGGCACAGACA-3', miR-214-R: 5'-AGTGCAGGGTCCGAGGTATT-3'; CDC25B-F: 5'-GCCTTCCTCCTACAGACAGT-3', CDC25B-R: 5'-AACTTATCCACGATGTTGCTGAA-3'; β-actin-F: 5'-GAGAAAATCTGGCACCACACC-3', β-actin-R: 5'-GGATAGCACAGCCTGGATAGCAA-3'; U6-F: 5'-CGCTTCGGCAGCACATATAC-3', U6-R: 5'-CAGGGGCCATGCTAATCTT-3'.

### Cell transfection

Three small interfering RNA (siRNA) sequences targeting FAM83A-AS1 and corresponding negative control sequence, along with miR-214 inhibitor and control were generated from GenePharma (Shanghai, China). KYSE30 and EC109 cells in the logarithmic growth phase were cultured in a six-well plate at a suitable density before transfection. Then cells were treated with siRNA or miRNA inhibitor with Lipofectamine 3000 reagents (Invitrogen) according to the manufacturer's instruction. The cells were cultured for 48 h at 37 °C after transfection for further assays. The sequences of si-FAM83A-AS1 and miR-214 inhibitor were listed in the following descriptions: si-FAM83A-AS1#1 sense: 5'-GCUGCCACCUACAAGAUAATT-3', anti-sense: 5'-UUAUCUUGUAGGUGGCAGCTT-3'; si-FAM83A-AS1#2 sense: 5'-GGCCCUGGGCUGAAUAAUUTT-3', antisense: 5'-AAUUAUUCAGCCCAGGGCCTT-3'; si-FAM83A-AS1#3 sense: 5'-CAGCCCUUCAGUGUUGAAATT-3', antisense: 5'-UUUCAACACUGAAGGGCUGTT-3'; si-NC sense: 5'-UUCUCCGAACGUGUCACGUTT-3', anti-sense: 5'-ACGUGACACGUUCGGAGAATT-3'; miR-214 inhibitor: 5'-ACUGCCUGUCUGUGCCUGCUGU-3'; miR-214 inhibitor NC: 5'-CAGUACUUUUGUGUAGUACAA-3'.

### Cell viability assay

We measured the cell proliferation rate by CCK-8 kit as previously described. ESCC cells treated with siRNAs were collected and seeded into a 96-well plate at a density of 2000 cells/well. Next, we added 10 μl CCK-8 reagent into each well after culturing for 24, 48, 72, 96 h. We detected the absorbance at 450 nm after incubation CCK-8 reagent for 2 h at 37 °C. Data were collected by a microplate reader for statistical analysis.

### Transwell assays

For the transwell migration and invasion assays, the upper chamber was coated with or without the matrigel. Subsequently, 2×10^4^ cells were seeded in the upper chamber containing 200 μl serum-free DMEM. In contrast, the 650 μl DMEM supplementing 15% FBS was added into the lower chamber carefully. After incubation for 24-48 h, those cells which did not migrate into the undersurface in the upper chamber were cleared away. Cells were fixed with 4% paraformaldehyde, stained with 0.1% crystal violet, and imaged with the microscope.

### Flow cytometry

The cell cycle and apoptosis analysis kit was utilized to detect the cell cycle distribution and apoptosis, respectively. For cell cycle assay, 5×10^6^ ESCC cells were digested with trypsin and washed by PBS. Then we fixed cells by pre-chilled 70% ethanol overnight at 4 °C. On the next day, we washed cells with PBS and acquired single-cell suspensions. Propidium iodide (PI) was used to stain the detached cells. Finally, we tested the cell cycle with the BD Accuri™ C6 Plus system and analyzed it with Modfit 4.0. For apoptosis assay, single-cell suspensions were labeled with FITC/PI double staining for 15 min at room temperature (25 °C). Subsequently, we used flow cytometry to detect early and late apoptosis of ESCC cells. FlowJo was used to analyze the percentages of apoptosis events.

### Statistical analysis

All data in the current study were displayed as mean ± standard deviation (S.D.). Student's t-test and Chi-square analysis were employed to examine the differences between the two groups according to the data types. One-way analyses of variance were adopted to analyze the differences between serval groups. Kaplan-Meier and ROC (Receiver operating characteristic curve) analysis were utilized to analyze the survival status and specificity and sensitivity, respectively. Pearson Correlation Coefficient was used to calculate the association. All statistical analyses were performed in GraphPad prism 8. *P<*0.05 was defined as statistical significance.

## Results

### FAM83A-AS1 is up-regulated in ESCC tissues

**Figure 1:**
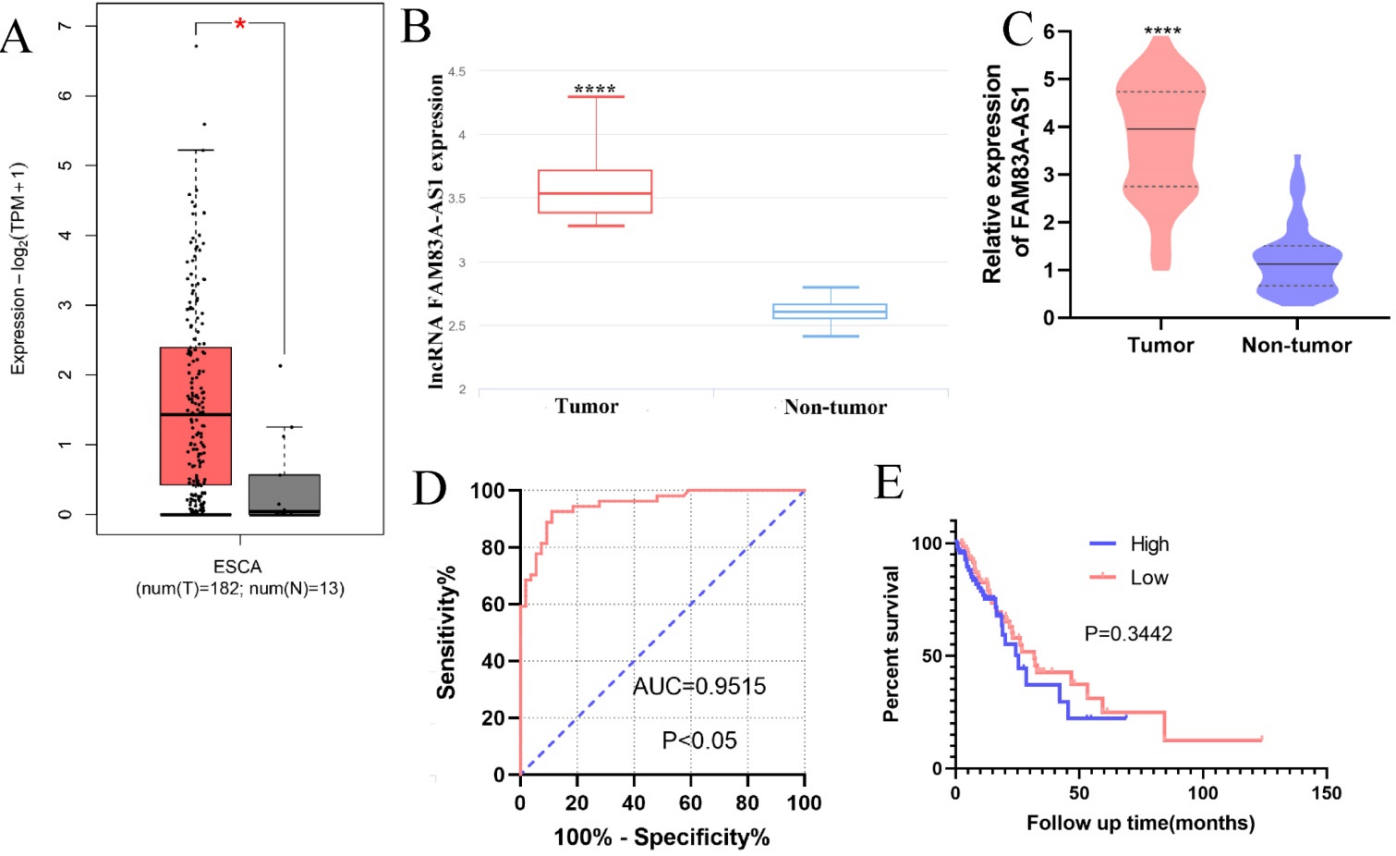
** FAM83A-AS1 was strongly overexpressed in ESCC tissues. (A-B)** The bioinformatic analysis retrieved from GEPIA and lnCAR illuminated that FAM83A-AS1 was accumulated in ESCC tissues (ESCA indicates the esophageal carcinoma). **(C)** FAM83A-AS1 was significantly increased in 51 ESCC tissues compared with the adjacent tumor tissues. **(D)** ROC analysis showed a high accuracy of FAM83A-AS1 in ESCC diagnosis. **(E)** Kaplan-Meier analysis revealed the association between FAM83A-AS1 expression and survival time of ESCC patients. *P<0.05, ****P<0.0001.

The bioinformatics data analysis retrieved from GEPIA (http://gepia2.cancer-pku.cn/#index) and lnCAR 16 revealed that FAM83A-AS1 was remarkably increased in ESCC (Fig. 1A-B). Consistently, our qRT-PCR results also corroborated the high expression of FAM83A-AS1 in 51 paired ESCC tissues compared with adjacent non-tumor tissues (Fig. 1C). Next, we collected the clinical information of these independent ESCC cases and assessed the relationships between FAM83A-AS1 level and clinical parameters of ESCC patients. We identified FAM83A-AS1 expression was significantly correlated with differentiation grade (*P=*0.0209, Table 1) and the advanced stages (*P=*0.0104, Table 1) of ESCC patients. However, there was no obvious association in other clinical parameters, including gender, age, and so on. Additionally, we assessed the diagnostic and prognostic effects of FAM83A-AS1 in ESCC. Kaplan-Meier curves suggested that patients with high expression of FAM83A-AS1 exhibited a similar overall survival time with those with low FAM83A-AS1 levels (Fig. 1E), whereas ROC analysis showed that FAM83A-AS1 gained high accuracy in distinguishing ESCC (Fig. 1D). The AUC (Area under the curve) was 0.954 (95% confidence intervals 0.895-0.985, CI) and sensitivity and specificity were 92.59% and 90.57%, respectively. Taken together, these findings implied that FAM83A-AS1 played a tumorigenic role in ESCC development and progression.

### FAM83A-AS1 accelarates ESCC cell growth

**Figure 2:**
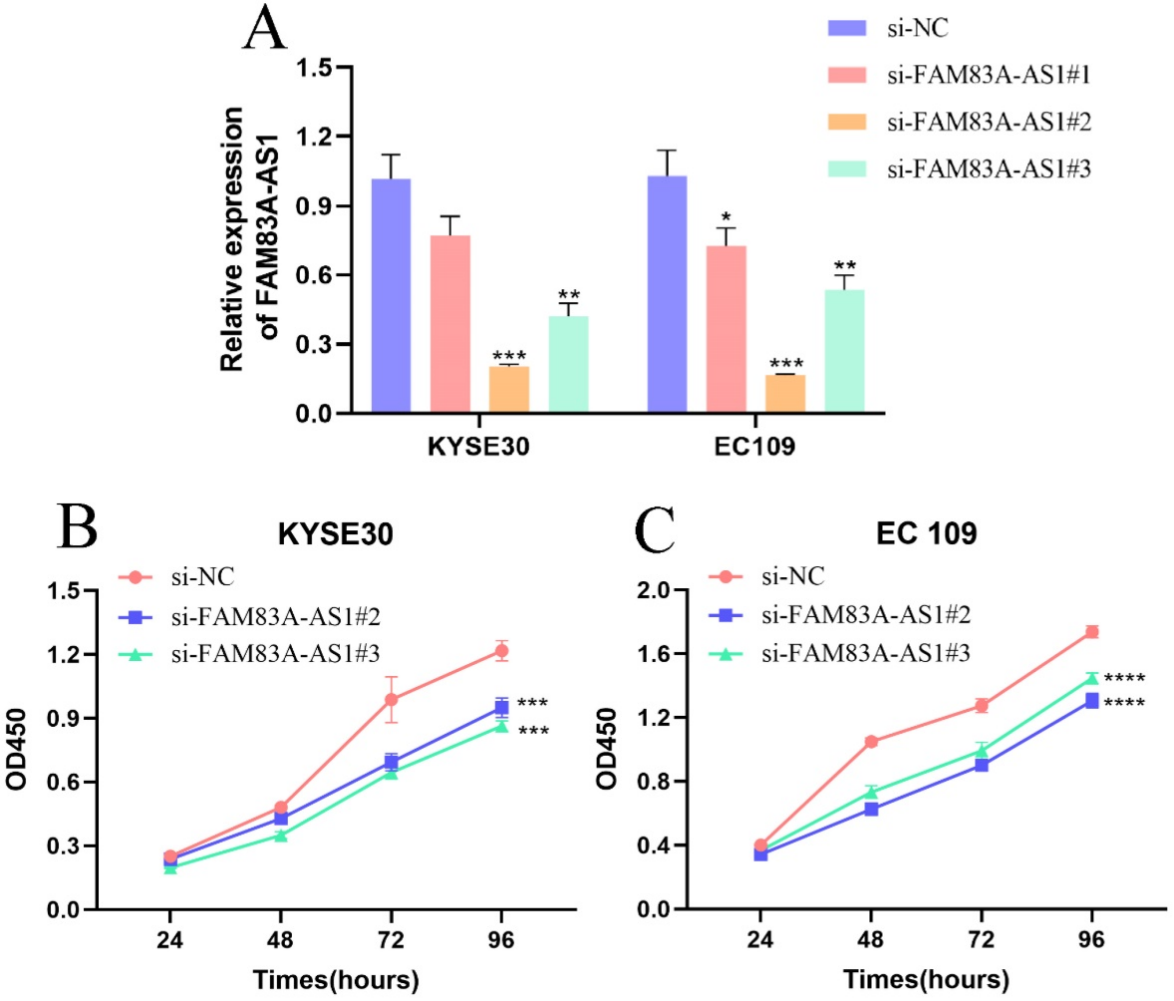
** Inhibiting FAM83A-AS1 impeded ESCC cell proliferation. (A)** The treatment of si-FAM83A-AS1 in KYSE30 and EC109 cells hampered FAM83A-AS1 expression. **(B-C)** CCK-8 assay results demonstrated that FAM83A-AS1 promoted ESCC cell generation. *P<0.05, **P<0.01, ***P<0.001, ****P<0.0001.

To evaluate the precise function of FAM83A-AS1 in ESCC cells, we designed three siRNAs targeting FAM83A-AS1 and a negative control sequence and named them as si-FAM83A-AS1#1, si-FAM83A-AS1#2, si-FAM83A-AS1#3, and si-NC. Next, we measured the gene silencing efficiency by qRT-PCR assays in KYSE30 and EC109 cells. We noticed that si-FAM83A-AS1#2 and si-FAM83A-AS1#3 exhibited higher gene silencing efficiency (Fig. 2A). Consequently, we focused on these two siRNAs for the following functional experiments. CCK-8 results demonstrated that cell proliferation ability was dramatically blunted after depleting FAM83A-AS1 in KYSE30 and EC109 cells (Fig. 2B-C). These data suggested that FAM83A-AS1 contributed to cell proliferation of ESCC cells *in vitro*.

### FAM83A-AS1 enhances ESCC cells metastasis capabilities

**Figure 3:**
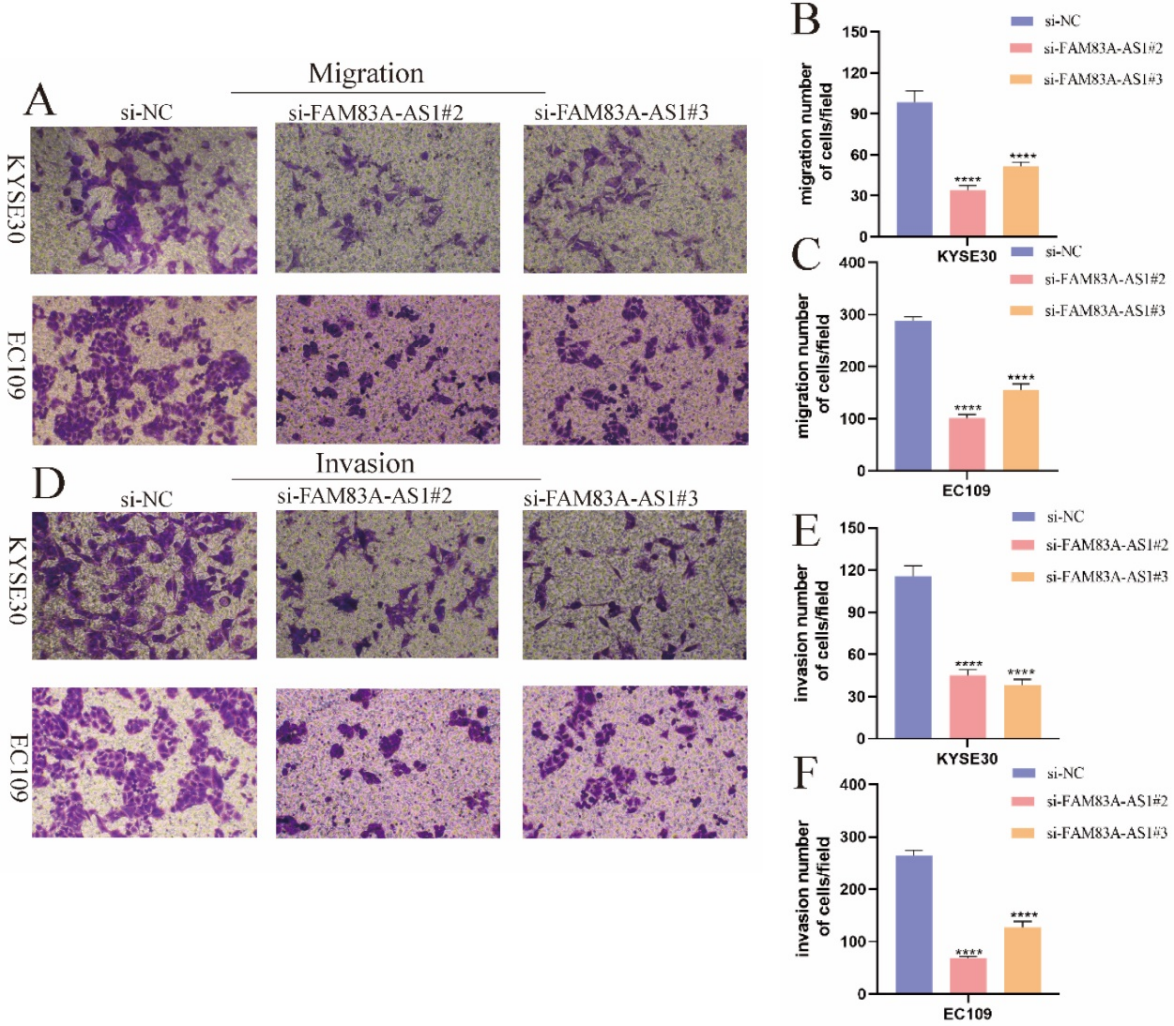
** FAM83A-AS1 increased ESCC cell metastasis. (A-C)** FAM83A-AS1 depletion suppressed cell migration activity in KYSE30 and EC109 cells. **(D-F)** The invasive ability was significantly reduced after knocking down FAM83A-AS1 in KYSE30 and EC109 cells. ****P<0.0001.

Given the importance of FAM83A-AS1 in ESCC, we further assessed the effect of FAM83A-AS1 on ESCC cell migration and invasion. Transwell migration and invasion assays were performed according to the previously described 17. Interestingly, we observed that KYSE30 cell migration capacity was significantly attenuated following knocking down FAM83A-AS1. Similarly, the migration assays in EC109 cells showed the same results (Fig. 3A-C). Furthermore, the transwell invasion assays indicated that the invasive capabilities of both KYSE30 and EC109 cells exhibited a decreasing trend in si-FAM83A-AS1 compared to the si-NC group, which was consistent with the migration assays. Overall, these investigations revealed that FAM83A-AS1 promoted ESCC metastasis process.

### FAM83A-AS1 regulates ESCC cells apoptosis events

**Figure 4:**
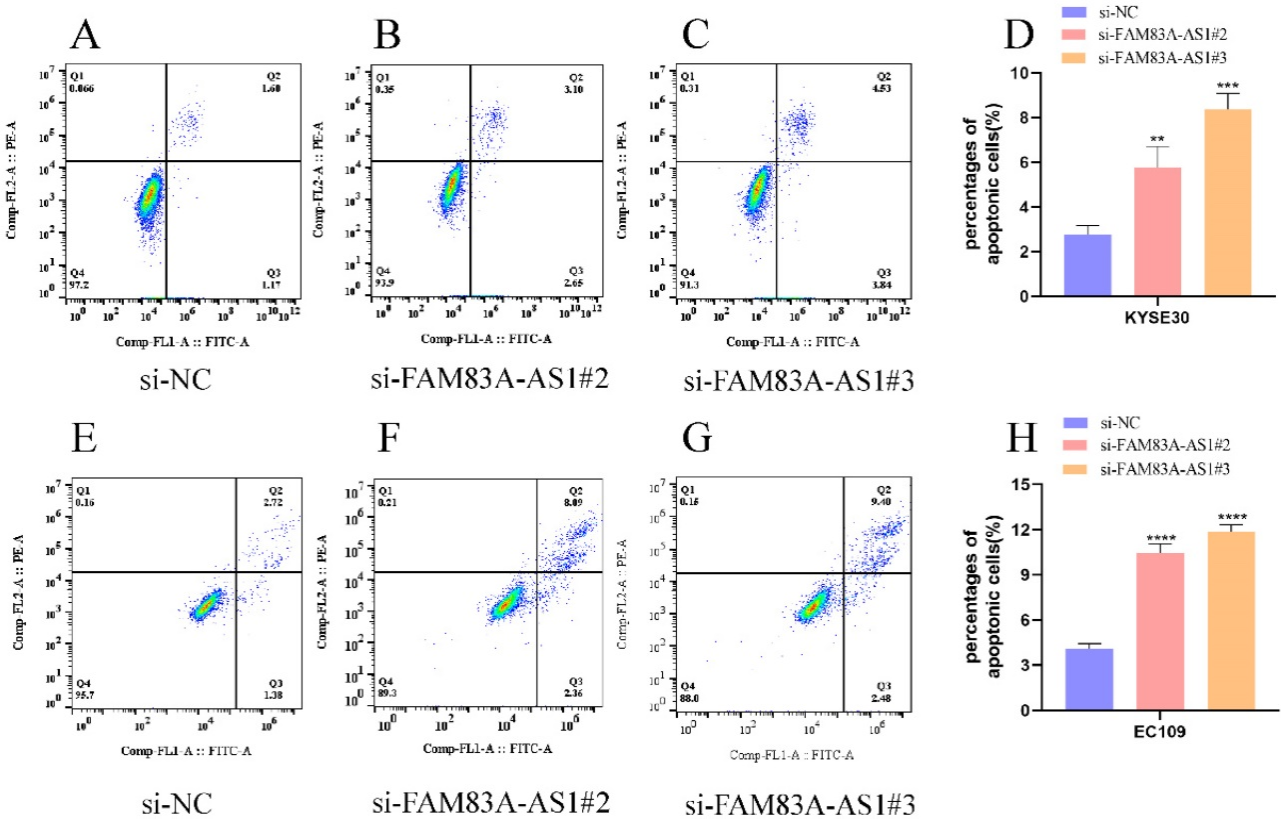
** FAM83A-AS1 negatively regulated cell apoptosis in ESCC. (A-D)** Flow cell cytometry was used to examine the apoptosis events in ESCC cells with different treatments. The total number of apoptosis cells was growing after inhibiting FAM83A-AS1 in KYSE30 cell. **(E-H)** Flow cell cytometry analysis based on EC109 treated with si-FAM83A-AS1 produced the resembling results. *P<0.05, **P<0.01, ***P<0.001, ****P<0.0001.

Annexin V/PI double staining assay was adopted to evaluate the influence of FAM83A-AS1 on ESCC cells' apoptosis progress. Correspondingly, we examined apoptosis events in ESCC cells using flow cell cytometry. Notably, we discovered that the percentage of cells in KYSE30 cells had an increase in KYSE30 cells after FAM83A-AS1 depletion (Fig. 4A-D). Meanwhile, inhibiting FAM83A-AS1 also led to the elevation of the proportion of apoptosis cells in EC109 cells (Fig. 4E-H). From these findings, we concluded that FAM83A-AS1 regulated ESCC cell apoptosis.

### FAM83A-AS1 expediates ESCC cells cycle distribution

**Figure 5:**
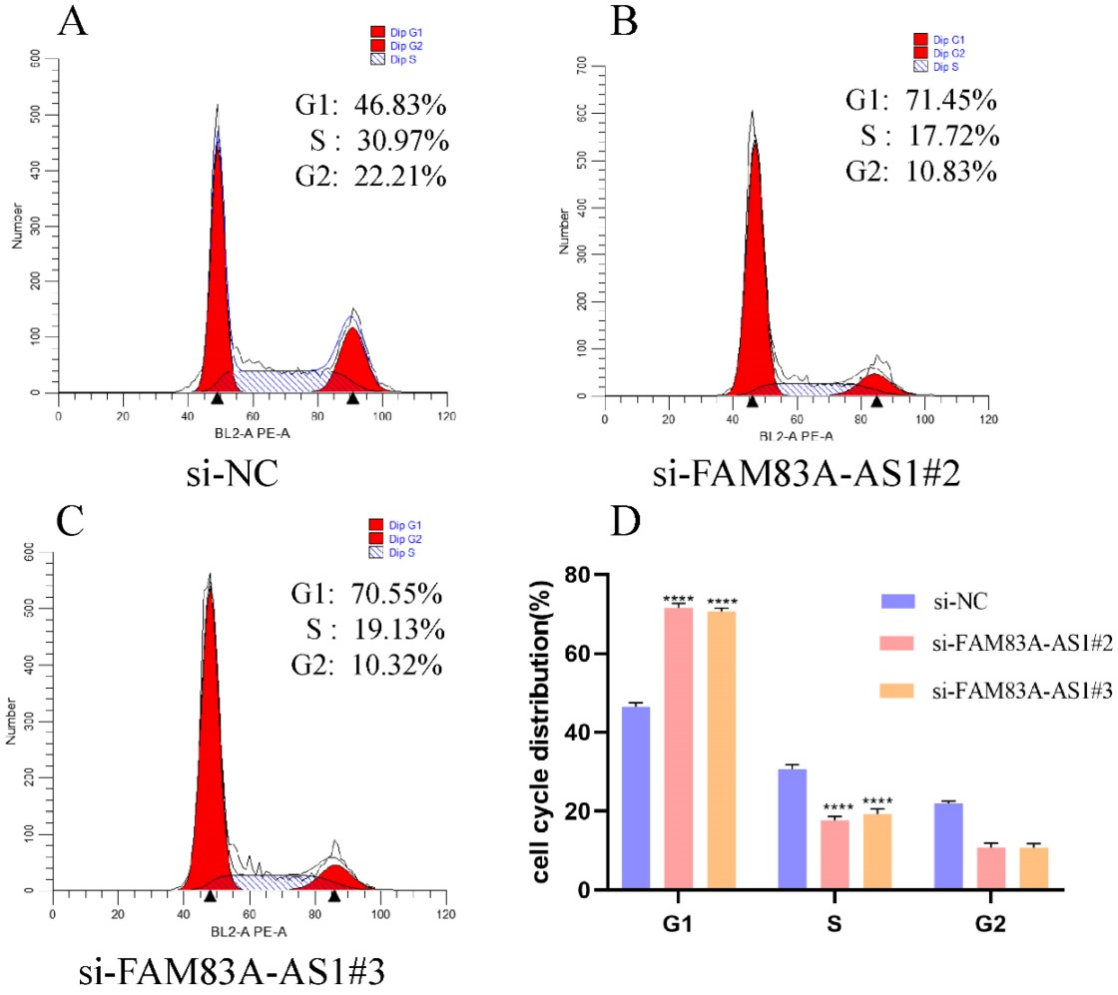
** FAM83A-AS1 regulated cell cycle distribution in ESCC. (A-D)** EC109 cell cycle distribution with flow cell cytometry was tested according to the instructions of the cell cycle detection kit. There was a remarkable rise of cells in the G1 phase while the count of cells in the S phase was reduced. ***P<0.001, ****P<0.0001.

To explore the potential mechanism of FAM83A-AS1 in promoting ESCC cell proliferation, we performed cell cycle assays based on PI staining with flow cell cytometry. The analysis results illustrated that FAM83A-AS1 remarkably regulated cell cycle distribution. Specifically, the population of G1 phase cells was strikingly augmented in the FAM83A-AS1-deficient group compared with the control group. In contrast, the cells in the S phase were sharply reduced after the silencing of FAM83A-AS1 (Fig. 5A-D). Taken together, these observations suggested that FAM83A-AS1 promoted cell proliferation through accelerating cell cycle process.

### FAM83A-AS1 regulates miR-214 expression pattern in ESCC cells

**Figure 6:**
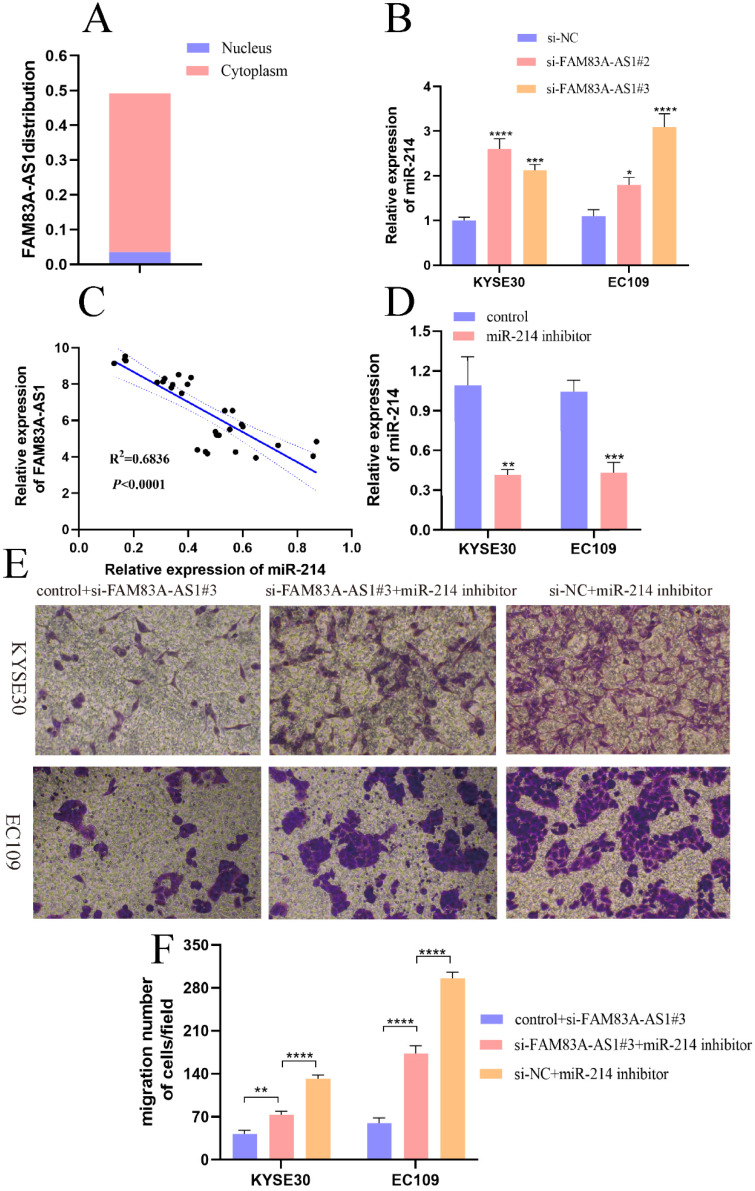
** FAM83A-AS1 promoted ESCC development by regulating miR-214. (A)** The subcellular localization of FAM83A-AS1 in the cell. **(B)** FAM83A-AS1 silencing produced an increase of miR-214 in ESCC cells. **(C)** The miR-214 expression pattern was negatively related to FAM83A-AS1 in ESCC tissues. **(D)** miR-214 inhibitor repressed miR-214 expression in ESCC cell. **(E-F)** The weakening of cell migration in ESCC cells induced by FAM83A-AS1 depletion could be reversed by miR-214 inhibitor partly. *P<0.05, **P<0.01, ***P<0.001, ****P<0.0001.

It was conclusively established that lncRNA could act as competing endogenous RNA by binding miRNAs, leading to the liberation of target mRNAs 18,19. First, we analyzed the location of FAM83A-AS1 in cells through lncLocator database (http://www.csbio.sjtu.edu.cn/bioinf/lncLocator/). Results indicated that FAM83A-AS1 was mainly accumulated in the cytoplasm (Fig. 6A). Moreover, we predicted potential miRNA candidates of FAM83A-AS1 by the Annolnc website. Here we found that miR-214 was a target miRNA of FAM83A-AS1. Additionally, it was reported that miR-214 suppressed ESCC progression by regulating cell proliferation, migration, and invasion 20. Therefore, we speculated that FAM83A-AS1 could regulate miR-214 expression to promote ESCC progression. As illustrated in Fig. 6B, there was an obvious increase of miR-214 expression level after FAM83A-AS1 depletion in KYSE30 and EC109 cells. Subsequently, miR-214 expression was negatively correlated with FAM83A-AS1 in ESCC tissues (Fig. 6C, R^2^=0.6836, *P<*0.0001). To confirm the role of miR-214 in ESCC cells, we designed the miR-214 inhibitor and the corresponding control sequence. As depicted in Fig. 6D, miR-214 expression was decreased after transfected with miR-214 inhibitor compared with the control group. Interestingly, we noted that cell migration potential was increased after FAM83A-AS1 knockdown combined with miR-214 inhibition compared with FAM83A-AS1 depletion (Fig. 6E-F). Collectively, these results illuminated that miR-214 could partially rescue the inhibition of cell migration caused by FAM83A-AS1 silencing. FAM83A-AS1 drives ESCC progression by regulating miR-214.

### The role of CDC25B in ESCC and the association between CDC25B, miR-214, and FAM83A-AS1

**Figure 7:**
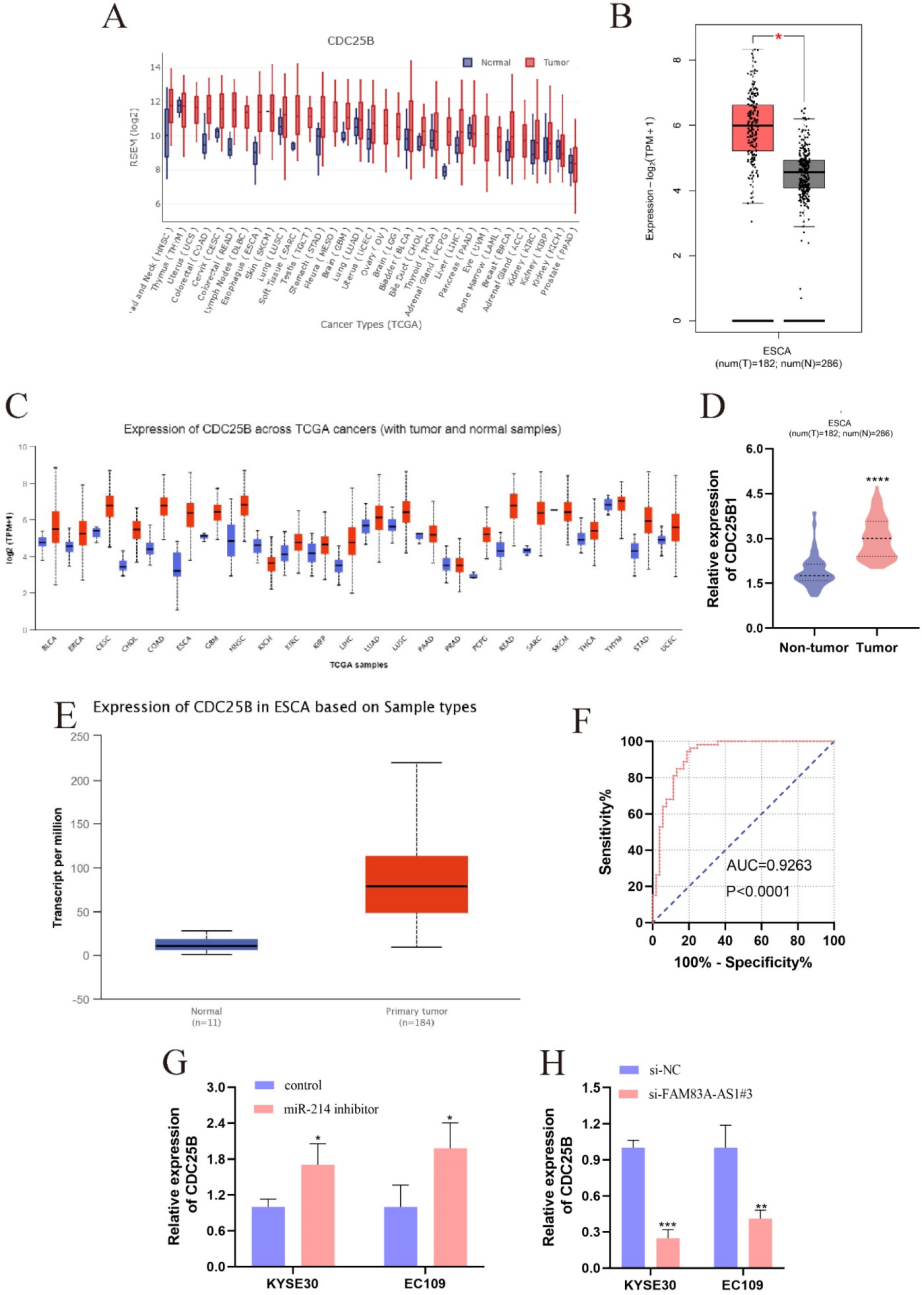
** The high expression of CDC25B in ESCC and the regulations between CDC25B, miR-214, and FAM83A-AS1. (A)** The pan-cancer analysis of CDC25B from GEDS. **(B)** CDC25B was significantly over-expressed in ESCC tissues, which was validated by GEPIA. **(C)** The pan-cancer analysis of CDC25B from Uclcan. **(D-E)** The up-regulation of CDC25B in ESCC was confirmed by oncomine and Uclcan. **(F)** The diagnostic value of CDC25B is based on the ROC analysis in ESCC. **(G)** miR-214 inhibitor engendered the augment of CDC25B in ESCC cells. **(H)** FAM83A-AS1 knockdown caused the reduction of CDC25B expression in ESCC cells. *P<0.05, **P<0.01, ***P<0.001, ****P<0.0001.

CDC25B was reported to be the direct target of miR-214 21. CDC25B was dramatically elevated in multiple cancers, which was validated by the pan-cancer analysis from GEDS 22 and Uclcan 23 (Fig. 7A, 7C). Notably, the CDC25B expression level was significantly higher in ESCC tissues. Meanwhile, data retrieved from GEPIA supported that CDC25B was predominantly accumulated in ESCC tissues compared to normal esophagus tissues (Fig. 7B). Bioinformatic analysis based on Uclcan and Oncomine (www.omcomine.org) also revealed that CDC25B was up-regulated in ESCC tissues (Fig. 7D-E). These investigations provided compelling evidence that CDC25B was an important driving force in ESCC development. Moreover, we found that CDC25B obtained a high diagnostic accuracy for ESCC (Fig. 7F, AUC=0.9263, *P<*0.0001). Interestingly, the CDC25B expression pattern was increased after inhibiting miR-214 in ESCC cells (Fig. 7G). In contrast, the CDC25B level exhibited a decreasing trend when ESCC cells were treated with si-FAM83A-AS1 (Fig. 7H). In summary, FAM83A-AS1 upregulated CDC25B expression by regulating miR-214 as molecular sponge.

## Discussion

Recently, considerable evidence in the literature have pointed out lncRNA is responsible for multiple cancer progression 24,25. It was widely observed that the ectopic expression pattern of lncRNAs occurred in tumor tissues and cells. More importantly, the dysregulation of lncRNAs was often associated with some clinical features, such as tumor size, lymphatic metastasis, and tumor stage 25. For example, SNHG3 was significantly dysregulated in thyroid carcinoma 26, lung cancer 27 and gastric cancer 28. The PVT1 expression level was associated with TNM stages, differentiation grade, and distant metastasis in gallbladder carcinoma 29. Moreover, lncRNAs exhibited great potential to be the candidate of biomarkers for diagnostic and prognosis evaluation of cancer. For instance, DLEU1 expression was correlated with the survival time of colorectal cancer patients 30. These pieces of evidence indicated lncRNA may play an oncogenic or tumor-suppressing role in different tumors. Specifically, they can serve as the key regulators of cancer-related signal pathways to control the biological function and cellular process. Hu et al. manifested that HOXC-AS3 promoted gastric cancer cell proliferation and metastasis by interacting with YBX1 at the transcriptional level 31. However, the expression pattern and molecular mechanism of many lncRNAs in ESCC remains elusive.

Here, we first defined the expression pattern of FAM83A-AS1 in ESCC. FAM83A-AS1 was strikingly elevated in ESCC tissues and the FAM83A-AS1 level was tightly associated with differentiation grade and advanced stages. In addition, FAM83A-AS1 showed great potential to be the biomarkers for ESCC diagnosis. However, there was no evident relationship between FAM83A-AS1 and survival time. More following-up investigations and survival data will be needed to definite it. Our research demonstrated that FAM83A-AS1 promoted cell proliferation, migration, and invasion in ESCC. Moreover, flow cell cytometry analysis showed that inhibiting FAM83A-AS1 led to the remarkable increase of cells in the S phase accompanied by the decrease of cells in the G1 phase, which indicated that FAM83A-AS1 promoted cell proliferation by expediating cell cycle progression. Notably, we noticed that the apoptosis events had progressively increased after knocking down FAM83A-AS1 in ESCC cells. Collectively, our work demonstrated that FAM83A-AS1 is a pivotal driver during ESCC development.

Accumulating evidence documented that lncRNA executed their function through diverse mechanisms including ceRNA, leading to the abnormal expression of the mRNAs 32. Binding with miR-let-7 in the cell cytoplasm, lncRNA GSTM3TV2 served as a ceRNA to up-regulate LAT2 and OLR1 expression, promoting cell gemcitabine resistance in pancreatic cancer. In the current study, we found that miR-214 was a direct target miRNA of FAM83A-AS1 in ESCC 33. It was reported that miR-214 inhibited cell proliferation, migration, and invasion process in ESCC 20. We discovered that there was a negative relationship between FAM83A-AS1 and miR-214 expression in ESCC tissues. And miR-214 expression was significantly elevated after knocking down FAM83A-AS1 in ESCC cells, which suggested that FAM83A-AS1 regulated miR-214 expression in ESCC. In terms of function, miR-214 inhibition partly comprised the ablation of cell migration activity induced by FAM83A-AS1 silencing. Combined with the current research investigations, we concluded that FAM83A-AS1 promoted ESCC progression by regulating miR-214. However, the luciferase reporter assay will be needed to confirm the exact binding site between FAM83A-AS1 and miR-214.

Given the regulation between miR-214 and FAM83A-AS1, we were determined to identify the downstream target genes of miR-214 to get a deeper understanding of the molecular mechanism of FAM83A-AS1 in ESCC. CDC25B was reported to be a direct target mRNA of miR-214 and CDC25B was strongly expressed in various cancers including ESCC 21. It was evident that CDC25B was an important oncogene to speed up cancer progression 34. CDC25B was significantly elevated in sera and cell lines of ESCC, and there was a strong relationship between CDC25B expression and prognosis of patients with advanced ESCC 35. In ovarian cancer, CDC25B inhibitor, WG-391D blunted cell proliferation, migration, resulting in cell cycle arrest at the G2/M and apoptosis 36. Herein, we discovered that CDC25B was significantly higher in ESCC tissues and CDC25B could serve as an independent biomarker in ESCC diagnosis. These analyses disclosed that the tumor-promoting effects of CDC25B in ESCC development. Notably, we found that FAM83A-AS1 knockdown impeded CDC25B expression. In contrast, CDC25B was strikingly increased in the ESCC cells treated with miR-214 inhibitor. Considering the regulation of FAM83A-AS1 on miR-214, we concluded that FAM83A-AS1 promoted ESCC development by regulating the miR-214/CDC25B axis for the first time.

## Conclusion

Taken together, our study revealed that FAM83A-AS1 exerted the oncogenic effects in ESCC through enhancing cell growth and metastasis while inhibiting cell apoptosis. We identified the regulatory mechanism of the FAM83A-AS1/miR-214/CDC25B axis in ESCC development and progression. These investigations suggested that FAM83A-AS1 might be a promising biomarker for ESCC diagnosis and therapy.

## Figures and Tables

**Figure 8:**
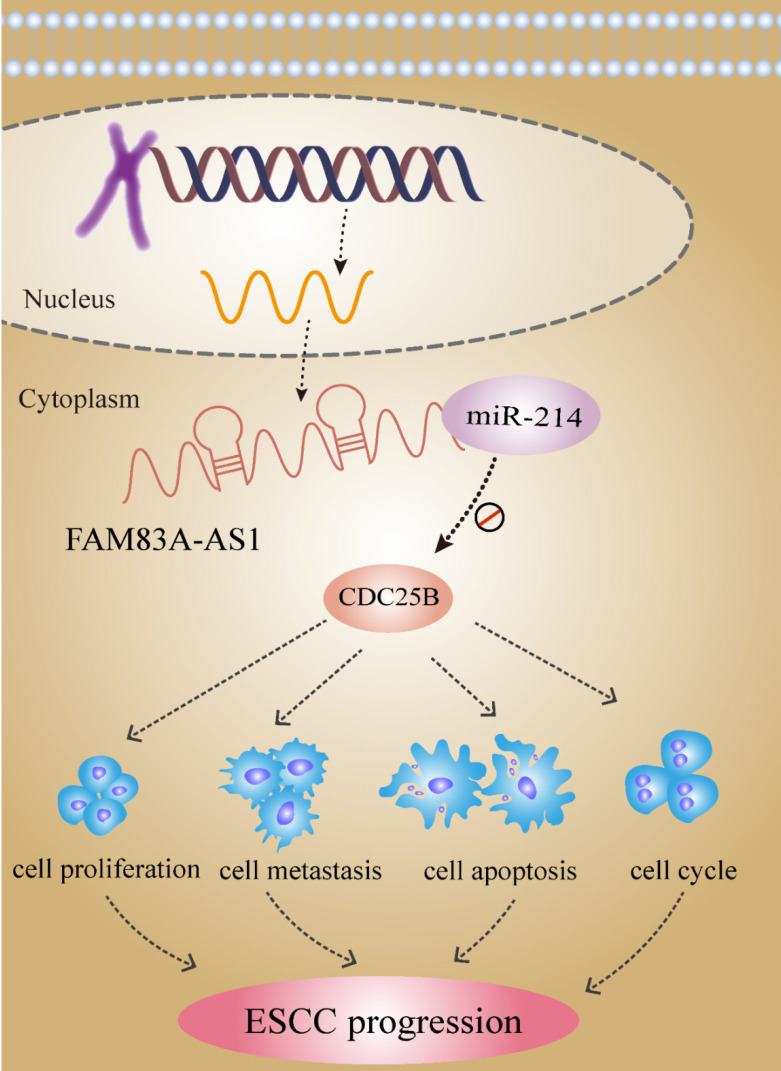
lncRNA FAM83A-AS1 dives ESCC progression by regulating the miR-214/CDC25B axis.

**Table 1:** Relationship between FAM83A-AS1 and pathological data in 51 ESCC patients

Variable	Number	FAM83A-AS1 expression		*P*-value
		High (26)	Low (25)	
**Gender**				
Male	21	13	8	0.1917
Female	30	13	17	
**Age**				
≤ 65 years	24	10	14	0.2097
> 65 years	27	16	11	
**Tumor size**				
≤ 3 cm	15	7	8	0.6908
> 3 cm	36	19	17	
**Differentiation grade**				
Moderate	40	17	23	0.0209*
Poor	11	9	2	
**TNM stage**				
I+II	39	16	23	0.0104*
III + IV	12	10	2	
**Lymphatic metastasis**				
Negative	27	13	14	0.6678
Positive	24	13	11	

*P < 0.05.

## References

[1] Chen Y, Lu Y, Ren Y, Yuan J, Zhang N, Kimball H (2020). Starvation-induced suppression of DAZAP1 by miR-10b integrates splicing control into TSC2-regulated oncogenic autophagy in esophageal squamous cell carcinoma. Theranostics.

[2] Teng H, Xue M, Liang J, Wang X, Wang L, Wei W (2020). Inter- and intratumor DNA methylation heterogeneity associated with lymph node metastasis and prognosis of esophageal squamous cell carcinoma. Theranostics.

[3] Fong L, Taccioli C, Palamarchuk A, Tagliazucchi G, Jing R, Smalley K (2020). Abrogation of esophageal carcinoma development in miR-31 knockout rats. Proceedings of the National Academy of Sciences of the United States of America.

[4] Liu K, Xie F, Zhao T, Zhang R, Gao A, Chen Y (2020). Targeting SOX2 Protein with Peptide Aptamers for Therapeutic Gains against Esophageal Squamous Cell Carcinoma. Molecular therapy: the journal of the American Society of Gene Therapy.

[5] Sun K, Zhao X, Wan J, Yang L, Chu J, Dong S (2018). The diagnostic value of long non-coding RNA MIR31HG and its role in esophageal squamous cell carcinoma. Life Science.

[6] Yang L, Sun K, Chu J, Qu Y, Zhao X, Yin H (2018). Long non-coding RNA FTH1P3 regulated metastasis and invasion of esophageal squamous cell carcinoma through SP1/NF-kB pathway. Biomedicine & Pharmacotherapy.

[7] Yang L, Ye Y, Chu J, Jia J, Qu Y, Sun T (2019). Long noncoding RNA FEZF1-AS1 promotes the motility of esophageal squamous cell carcinoma through Wnt/beta-catenin pathway. Cancer Management Research.

[8] Xie S, Zhang J, Jiang X, Hua Y, Xie S, Qin Y (2020). LncRNA CRNDE facilitates epigenetic suppression of CELF2 and LATS2 to promote proliferation, migration and chemoresistance in hepatocellular carcinoma. Cell death & disease.

[9] Chen Q, Li B, Liu D, Zhang B, Yang X, Tu Y (2020). LncRNA KCNQ1OT1 sponges miR-15a to promote immune evasion and malignant progression of prostate cancer via up-regulating PD-L1. Cancer cell international.

[10] Sun X, Qian Y, Wang X, Cao R, Zhang J, Chen W (2020). LncRNA TRG-AS1 stimulates hepatocellular carcinoma progression by sponging miR-4500 to modulate BACH1. Cancer cell international.

[11] Chu J, Li H, Xing Y, Jia J, Sheng J, Yang L (2019). LncRNA MNX1-AS1 promotes progression of esophageal squamous cell carcinoma by regulating miR-34a/SIRT1 axis. Biomedicine & Pharmacotherapy.

[12] Cheng Z, Wang G, Zhu W, Luo C, Guo Z (2020). LEF1-AS1 accelerates tumorigenesis in glioma by sponging miR-489-3p to enhance HIGD1A. Cell death & disease.

[13] Shi R, Jiao Z, Yu A, Wang T (2019). Long noncoding antisense RNA FAM83A-AS1 promotes lung cancer cell progression by increasing FAM83A. Journal of cellular biochemistry.

[14] Xiao G, Wang P, Zheng X, Liu D, Sun X (2019). FAM83A-AS1 promotes lung adenocarcinoma cell migration and invasion by targeting miR-150-5p and modifying MMP14. Cell cycle (Georgetown, Tex.).

[15] He J, Yu J (2019). Long noncoding RNA FAM83A-AS1 facilitates hepatocellular carcinoma progression by binding with NOP58 to enhance the mRNA stability of FAM83A. Bioscience reports.

[16] Zheng Y, Xu Q, Liu M, Hu H, Xie Y, Zuo Z (2019). lnCAR: A Comprehensive Resource for lncRNAs from Cancer Arrays. Cancer research.

[17] Chu J, Jia J, Yang L, Qu Y, Yin H, Wan J (2020). LncRNA MIR31HG functions as a ceRNA to regulate c-Met function by sponging miR-34a in esophageal squamous cell carcinoma. Biomedicine & Pharmacotherapy.

[18] Zheng Z, Li Z, Zhou G, Lin L, Zhang L, Lv J (2019). Long Noncoding RNA FAM225A Promotes Nasopharyngeal Carcinoma Tumorigenesis and Metastasis by Acting as ceRNA to Sponge miR-590-3p/miR-1275 and Upregulate ITGB3. Cancer research.

[19] Li Y, Zeng C, Hu J, Pan Y, Shan Y, Liu B (2018). Long non-coding RNA-SNHG7 acts as a target of miR-34a to increase GALNT7 level and regulate PI3K/Akt/mTOR pathway in colorectal cancer progression. Journal of hematology & oncology.

[20] Huang S, Yuan Y, Zhuang C, Li B, Gong D, Wang S (2012). MicroRNA-98 and microRNA-214 post-transcriptionally regulate enhancer of zeste homolog 2 and inhibit migration and invasion in human esophageal squamous cell carcinoma. Molecular cancer.

[21] Wang M, Wang L, Zhang M, Li X, Zhu Z, Wang H (2017). MiR-214 inhibits the proliferation and invasion of esophageal squamous cell carcinoma cells by targeting CDC25B. Biomedicine & pharmacotherapy.

[22] Xia M, Liu C, Zhang Q, Guo A (2019). GEDS: A Gene Expression Display Server for mRNAs, miRNAs and Proteins. Cells.

[23] Chandrashekar D, Bashel B, Balasubramanya S, Creighton C, Ponce-Rodriguez I, Chakravarthi B (2017). UALCAN: A Portal for Facilitating Tumor Subgroup Gene Expression and Survival Analyses. Neoplasia (New York, N.Y.).

[24] Zhang W, Qin L, Wang J, Fan J, Lei C, Liu Q (2018). HOTAIR promotes proliferation, migration and invasion of esophageal squamous cell carcinoma by regulating MAPK1. International Journal of Clinical and Experimental Medicine.

[25] Zhang H, Cai Y, Zheng L, Zhang Z, Lin X, Jiang N (2018). Long noncoding RNA NEAT1 regulate papillary thyroid cancer progression by modulating miR-129-5p/KLK7 expression. Journal of Cellular Physiology.

[26] Duan Y, Wang Z, Xu L, Sun L, Song H, Yin H (2020). lncRNA SNHG3 acts as a novel Tumor Suppressor and regulates Tumor Proliferation and Metastasis via AKT/mTOR/ERK pathway in Papillary Thyroid Carcinoma. J Cancer.

[27] Shi J, Li J, Yang S, Hu X, Chen J, Feng J (2020). LncRNA SNHG3 is activated by E2F1 and promotes proliferation and migration of non-small-cell lung cancer cells through activating TGF-β pathway and IL-6/JAK2/STAT3 pathway. Journal of cellular physiology.

[28] Xuan Y, Wang Y (2019). Long non-coding RNA SNHG3 promotes progression of gastric cancer by regulating neighboring MED18 gene methylation. Cell death & disease.

[29] Chen J, Yu Y, Li H, Hu Q, Chen X, He Y (2019). Long non-coding RNA PVT1 promotes tumor progression by regulating the miR-143/HK2 axis in gallbladder cancer. Molecular Cancer.

[30] Liu T, Han Z, Li H, Zhu Y, Sun Z, Zhu A (2018). LncRNA DLEU1 contributes to colorectal cancer progression via activation of KPNA3. Mol Cancer.

[31] Zhang E, He X, Zhang C, Su J, Lu X, Si X (2018). A novel long noncoding RNA HOXC-AS3 mediates tumorigenesis of gastric cancer by binding to YBX1. Genome biology.

[32] Hua Q, Jin M, Mi B, Xu F, Li T, Zhao L (2019). LINC01123, a c-Myc-activated long non-coding RNA, promotes proliferation and aerobic glycolysis of non-small cell lung cancer through miR-199a-5p/c-Myc axis. Journal of hematology & oncology.

[33] Xiong G, Liu C, Yang G, Feng M, Xu J, Zhao F (2019). Long noncoding RNA GSTM3TV2 upregulates LAT2 and OLR1 by competitively sponging let-7 to promote gemcitabine resistance in pancreatic cancer. Journal of hematology & oncology.

[34] Takemasa I, Yamamoto H, Sekimoto M, Ohue M, Noura S, Miyake Y (2000). Overexpression of CDC25B phosphatase as a novel marker of poor prognosis of human colorectal carcinoma. Cancer research.

[35] Dong J, Zeng B, Xu L, Wang J, Li M, Zeng M (2010). Anti-CDC25B autoantibody predicts poor prognosis in patients with advanced esophageal squamous cell carcinoma. Journal of translational medicine.

[36] Xiao Y, Yu Y, Gao D, Jin W, Jiang P, Li Y (2019). Inhibition of CDC25B With WG-391D Impedes the Tumorigenesis of Ovarian Cancer. Frontiers in oncology.

